# C-853-03. Development of an Algorithm to Guide Early Mobility in the Burn ICU

**DOI:** 10.1093/jbcr/irag033.071

**Published:** 2026-04-06

**Authors:** Rachel B Gonzalez, Joanna Keough, Laura Goyack

**Affiliations:** Warden Burn Center at Orlando Health, Orlando Regional Medical Center, Orlando, FL; Warden Burn Center at Orlando Health, Orlando Regional Medical Center, Orlando, FL; Warden Burn Center at Orlando Health, Orlando Regional Medical Center, Orlando, FL

## Abstract

**Introduction:**

Over the past 20 years the importance of early mobility in the critically ill patient has been well defined in the literature. Prolonged immobility is associated with many adverse outcomes including intensive care unit (ICU) acquired weakness, decrease in muscle mass, loss of function, delirium, cognitive impairment and overall unfavorable outcomes. Additional considerations and barriers to mobilizing the critically ill burn patient include the use of vasopressors, continuous renal replacement therapy (CRRT), mechanical ventilation settings and recent surgical intervention. An article published by Lindholz et all in 2022 showed that mobilization out of bed on norepinephrine up to 0.20 ug/kg/min did not result in an increase in adverse events. Similar studies have been conducted without significant increase in adverse effects when mobilizing patients on CRRT. One article published in 2022 by O’Neil et al examined the use of an algorithm in the burn ICU for early mobilization of patients on mechanical ventilation. It defined progressive mobility in stages and looked at the levels of ICU mobility achieved. Since that time there have been studies updating recommendations on drips, lines and early mobility. This algorithm includes updated recommendations and the use of a valid outcome measure to help guide early mobility in the ICU.

**Methods:**

A delphi group of experts including attending physicians from several ICU’s, registered nurses, respiratory therapists, physical and occupational therapists all assisted in the development of this algorithm. The guideline includes critical checks for beginning mobility, hemodynamic parameters that indicate when progression is not appropriate, a breakdown of what level of mobility is appropriate from phase 0 to phase 2 based on the above checks, use of the ICU mobility scale (a valid tool for the progression of mobility in the ICU setting) and guidance on vasopressor and inotrope dosing as well as the use of CRRT with regards to level of mobility.

**Results:**

A new algorithm was created to help guide the ICU practitioner on mobilizing the critically ill patient. It looks at the whole patient and allows for systematic assessment by all providers.

**Conclusions:**

The creation of an updated early mobility algorithm integrates the latest evidence into a multidisciplinary guideline for safe mobilization of critically ill patients. It incorporates body systems, hemodynamic status, invasive tubes/lines, and medications to help the practitioner make informed decisions about mobilizing the critically ill patient.

**Applicability of Research to Practice:**

It is our hope that this algorithm will help increase comfort of the multidisciplinary team with mobilizing the critically ill patient by giving a set criteria for guidance and monitoring. Use of a valid outcome measure in the protocol and incorporation into the electronic medical record will allow for future tracking and data collection to assess the success of this tool.

**Funding for the study:**

N/A.

